# Association between single nucleotide polymorphisms within HLA region and disease relapse for patients with hematopoietic stem cell transplantation

**DOI:** 10.1038/s41598-019-50111-5

**Published:** 2019-09-24

**Authors:** Ding-Ping Chen, Su-Wei Chang, Po-Nan Wang, Fang-Ping Hus, Ching-Ping Tseng

**Affiliations:** 1 Department of Laboratory Medicine, Chang Gung Memorial Hospital, Taoyuan, Taiwan; 2 Department of Medical Biotechnology and Laboratory Science, College of Medicine, Chang Gung University, Taoyuan, Taiwan; 3 Graduate Institute of Biomedical Sciences, College of Medicine, Chang Gung University, Taoyuan, Taiwan; 4 Clinical Informatics and Medical Statistics Research Center, College of Medicine, Chang Gung University, Taoyuan, Taiwan; 5 Division of Allergy, Asthma, and Rheumatology, Department of Pediatrics, Chang Gung Memorial Hospital, Taoyuan, Taiwan; 6 Division of Hematology-Oncology, Department of Internal Medicine, Chang Gung Memorial Hospital, Taoyuan, Taiwan; 7 Molecular Medicine Research Center, Chang Gung University, Taoyuan, Taiwan

**Keywords:** Genetics research, Molecular medicine

## Abstract

Disease relapse occurs in patients with leukemia even hematopoietic stem cell transplantation (HSCT) was performed with human leukocyte antigen (HLA)-matched donors. As revealed previously by Petersdorf *et al*., there are nine single nucleotide polymorphisms (SNPs) located in the HLA region that potentially modulate the efficacy of HSCT. In this study, we investigated whether or not the genomic variants 500 base pairs flanking the nine transplantation-related SNPs were related to the risk of post-HSCT relapse for patients with leukemia (n = 141). The genomic DNAs collected from 85 patients with acute myeloid leukemia (AML), 56 patients with acute lymphocytic leukemia (ALL), and their respective HLA-matched donors were subject to SNPs analysis, conferred by the mode of mismatch between donor-recipient pair or by recipient or donor genotype analysis. Seven SNPs were revealed to associate with the risk of relapse post-HSCT. For patients with AML, the increased risk of post-HSCT relapse was associated with the donor SNP of rs111394117 in the intron of NOTCH4 gene, and the recipient SNPs of rs213210 in the ring finger protein 1 (RING1) gene promoter, and rs17220087 and rs17213693 in the intron of HLA-DOB gene. For patients with ALL, the increased risk of post-HSCT relapse was associated with the donor SNP of rs213210 in the RING1 gene promoter, and the recipient SNPs of rs79327197 in the HLA-DOA gene promoter, rs2009658 in the telomeric end of lymphotoxin-alpha (LTA) gene, rs17220087 and rs17213693 in the intron of HLA-DOB gene, and rs2070120 in the 3′-UTR of HLA-DOB gene. This study sheds new insight into selecting better candidate donors for performing HSCT in patients with AML and ALL.

## Introduction

The human leukocyte antigen (HLA) region located on chromosome 6p21.3 represents the most polymorphic region of the human genome. With the high density distribution of genes related to immune function, identification of clinically important genetic variants located in HLA region is crucial in stem cell transplantation^1^. Allogeneic hematopoietic stem cell transplantation (HSCT) is an approach to treat different types of hematologic disorders^2–5^. Patients with HSCT are able to receive high dose of therapeutic regimens to increase the cure rate. Disease relapse represents the major cause of transplant failure, while post-HSCT death is mainly caused by infection and graft-versus-host disease (GVHD). High risks of disease relapse and GVHD and high mortality are caused by transplantation of recipients with mismatched HLA donors when compared with HLA-matched donors^6,7^. The outcome of transplantation is usually better for related donor-recipient pairs when compared to the unrelated pairs^8,9^. Accordingly, related HLA-matched donors are the priority choice when allo-transplantation is performed.

Patient death may still occur even when HLA-matched donors are used in HSCT^10^. This implies that the outcomes of HSCT are likely related to other genetic factors in addition to the HLA alleles. A recent study conducted by Petersdorf *et al*. demonstrated that within the HLA region, there are nine single nucleotide polymorphisms (SNPs) (rs2244546, rs915654, rs429916, rs2242656, rs209130, rs2075800, rs394657, rs2071479 and rs107822) related to the occurrence of adverse effects associated with HSCT, including the patient death, transplant-related death, disease-free survival, relapse, and acute and chronic GVHD^11^. These SNPs in the form of donor DNA, recipient DNA, or mismatch between donor-recipient pair DNA lead to unfavorable or favorable outcome of patient post-HSCT^11^. These studies indicate that the efficacy of HSCT is affected by the genetic variants in the HLA region^12–14^.

We speculated that the relapse for patient with HLA-matched HSCT might be conferred by undefined genetic variants located at the HLA region of donor and recipient genome. The issue was investigated in this study by determining and analyzing the genomic sequences 500 base pair (bp) flanking the nine HSCT-related SNPs^11^. The significance of these findings in the strategic plan of HSCT is discussed.

## Results

Patients (n = 141) with acute myeloid leukemia (AML, n = 85) and acute lymphocytic leukemia (ALL, n = 56) receiving HSCT from HLA-matched donors were recruited to determine whether or not the risk of relapse is related to the SNPs within the HLA region (Table 1). Genomic DNA of the donor-recipient pairs were subject to PCR amplification of the genomic regions 500 bp flanking the 9 sourced SNPs (Table 2) using the forward and reversed primers (Table 3). Candidate SNPs that were related to the post-HSCT relapse were searched by sequencing the PCR amplicons. Collectively 34 SNPs in the HLA region were defined to associate with post-HSCT relapse. These SNPs were classified into group 1 (donor genotype, n = 5), group 2 (recipient genotype, n = 11), and group 3 (donor-recipient pair mismatch type, n = 18) SNPs according to the category of and the relative position to the sourced SNPs (Table 2). The association between these SNPs and the risk of disease relapse were analyzed and conferred by donor SNP (mode of donor genotype analysis), recipient SNP (mode of recipient genotype analysis) or mismatched of donor-recipient pair SNP (mode of mismatch between donor-recipient pair, defined by having a specific combination of different SNP alleles between the donor and recipient).

**Table 1:** Clinical characteristics of patients with AML and ALL receiving HSCT.

Characteristics of patients	Number of patient (%) or median (range)
Number of patients	141
Median age in years (range)	34 (4~64)
Male: Female	76 (53.9%): 65 (46.1%)
Diagnosis	
AML	85 (60.3%)
ALL	56 (39.7%)
Acute GVHD	105 (74.5%)
Grade I	30 (21.3%)
Grade II	43 (30.5%)
Grade III	20 (14.2%)
Grade IV	12 (8.5%)
Chronic GVHD	19 (13.5%)
No GVHD	17 (12.1%)
Overall survival	84 (59.6%)
Relapse	41 (29.1%)

**Table 2:** The SNPs that are 500 bps flanking the sourced SNPs*.

Sourced SNP	Model	SNP under analysis			
rs2244546	Donor genotype	rs2523675	rs2518028	rs141431529	
rs394657	Donor genotype	rs2256594	rs111394117		
rs429916	Recipient genotype	rs9276982	rs71565361	rs79327197	rs151190962
		rs9282369			
rs915654	Recipient genotype	rs2009658	rs736160	rs915654	
rs2075800	Recipient genotype	rs371621895	rs2075800	rs2227956	
rs2242656	Mismatch	rs3130048	rs2844464	rs2242656	
rs107822	Mismatch	rs107822	rs213210		
rs209130	Mismatch	rs209132	rs209131	rs209130	rs1536215
		rs139791445	rs6928948		
rs2071479	Mismatch	rs11244	rs2070120	rs41258084	rs17220087
		rs2071479	rs17213693	rs2070121	

*The table was partially reproduced from the reference^35^.

**Table 3:** The primer sequences for amplification of gene fragments by PCR*.

Gene	Primer Sequences
BAG6^#^	F: 5′-ATTCATTCAGGGGCACAAGGGG-3′ R: 5′-GCGGAGGTTGAAGAGAATAGAAGC-3′
HCP5	F: 5′-GGGCAACTAAGTCAGGTCTAG-3′ R: 5′-TCTGCAGGTCTCATGGAGAG-3′
HLA-DOA	F: 5′-CAACAACGTAAAGCTAACGTCTGTG-3′ R: 5′-GCACCACTCTTAGTTATGTATAGG-3′
HLA-DOB	F: 5′-TCTTCTGAAGACTGTGGAGACTGC-3′ R: 5′-TCCCATAGGAGCTCAGTCTGAAT-3′
HSPA1L	F: 5′-TCCCCTTCAAGGTACATTCACAGCC-3′ R: 5′-TGATCCAGGTGTATGAGGGCGAGAG-3′
LTA	F: 5′-AGCATAAAAGGCAAAGGGGCAG-3′ R: 5′-TTAGGTATGAGGTGGACACCTC-3′
NOTCH4	F: 5′-GATTGTCTGTTGGGTGACCTGAG-3′ R: 5′-TGAGGCTGATCACAATGAGTGCCTCTC-3′
RING1	F: 5′-TAATCGACTCTGGCGCCCACAT-3′ R: 5′-AACAACCTTAGCCTCGGTTCCCTT-3′
TRIM27	F: 5′-AGTCGGGATTACAGAAATGCACC-3′ R: 5′-GCAGGACATTTGAAGGTAACC-3′

*The table was partially reproduced from reference^35^.

^#^BAG6: BCL2 associated athanogene 6; HCP5: HLA complex P5; HLA-DOA: major histocompatibility complex, class II, DO alpha; HLA-DOB: major histocompatibility complex, class II, DO beta; HSPA1L: heat shock protein family A member 1 like; LTA: lymphotoxin alpha; RING1: ring finger protein 1; TRIM27: tripartite motif containing 27.

Of the 5 SNPs in group 1, three SNPs were located at the telomeric end of HLA class I histocompatibility antigen protein P5 (HCP5) gene and two SNPs were located in the intron of NOTCH4 gene, respectively. Donor genotype analysis demonstrated that none was related to the risk of relapse for patients with AML (Supplementary Table 1). However, the SNP of rs111394117 in the NOTCH4 intron was related to the risk of relapse for patients with ALL (genotypic test: *P* = 0.0166; Table 4). A greater risk of relapse for patients with ALL was associated with the donors who carried the polymorphism of A at rs111394117 when compared to the donors who carried the polymorphism of G at the same SNP position.

**Table 4:** The relapse-associated SNPs for patients with HSCT.

SNP	Genome position^1^ (bp)	Gene/location	Source^2^	Disease/Status	Number of patients (%)			P^*^
**Donor genotype**								
rs111394117	32219436	NOTCH4, intron	rs394657	AML	A/A	A/G	G/G	0.0166
				Relapse	1 (6.7)	2 (13.3)	12 (80.0)	
				Non-relapse	0 (0.0)	0 (0.0)	40 (100.0)	
rs213210	33208047	RING1, promoter	rs107822	ALL	A/A	A/G	G/G	0.0285
				Relapse	3 (25.0)	8 (66.7)	1 (8.3)	
				Non-relapse	10 (25.0)	12 (30.0)	18 (45.0)	
**Recipient genotype**								
rs79327197	33010635	HLA-DOA,	rs429916	ALL	A/A	A/G	G/G	0.0150
		promoter		Relapse	10 (66.7)	5 (33.3)	0 (0.0)	
				Non-relapse	35 (94.6)	2 (5.4)	0 (0.0)	
rs2009658	31538244	1.6 kb telomeric of	rs915654	ALL	C/C	C/G	G/G	0.0148
		LTA		Relapse	12 (80.0)	1 (6.7)	2 (13.3)	
				Non-relapse	25 (67.6)	12 (32.4)	0 (0.0)	
rs213210	33208047	RING1, promoter	rs107822	AML	A/A	A/G	G/G	0.0444
				Relapse	2 (8.3)	13 (54.2)	9 (37.5)	
				Non-relapse	20 (34.5)	26 (44.8)	12 (20.7)	
rs17220087	32813299	HLA-DOB, intron	rs2071479	AML	A/A	A/C	C/C	0.0465
				Relapse	0 (0.0)	0 (0.0)	25 (100.0)	
				Non-relapse	0 (0.0)	8 (14.3)	48 (85.7)	
				ALL	A/A	A/C	C/C	0.0311
				Relapse	0 (0.0)	3 (20.0)	12 (80.0)	
				Non-relapse	0 (0.0)	1 (2.6)	37 (97.4)	
rs17213693	32813344	HLA-DOB, intron	rs2071479	AML	C/C	C/G	G/G	0.0465
				Relapse	0 (0.0)	0 (0.0)	25 (100.0)	
				Non-relapse	0 (0.0)	8 (14.3)	48 (85.7)	
				ALL	C/C	C/G	G/G	
				Relapse	0 (0.0)	3 (20.0)	12 (80.0)	0.0311
				Non-relapse	0 (0.0)	1 (2.6)	37 (97.4)	
rs2070120	32813137	HLA-DOB, 3′UTR	rs2071479	ALL	A/A	A/G	G/G	0.0311
				Relapse	0 (0.0)	3 (20.0)	12 (80.0)	
				Non-relapse	0 (0.0)	1 (2.6)	37 (97.4)	

*Statistical analyses were performed by using genotypic test or Chi-square test.

Of the 11 SNPs in group 2, five SNPs were located at the HLA-DOA gene promoter, three SNPs were located at the telomeric end of lymphotoxin-alpha (LTA) gene, and three SNPs were located in the intron of the heat shock protein family A member 1 like (HSPA1L) gene, respectively. Recipient genotype analysis revealed that none was associated with the risk of relapse for patients with AML (Supplementary Table 2). On the other hand, the risk of relapse was associated with the SNPs of rs79327197 located at the HLA-DOA promoter and rs2009658 located at the telomeric end of LTA gene for patients with ALL (rs79327197: genotypic test, *P* = 0.015; rs2009658: genotypic test, P = 0.0148; Table 4). A greater risk of relapse was associated with the patients who had the polymorphism of G at rs79327197 and G at rs2009658 than the recipients who had the polymorphism of A and C at the corresponding position of SNP, respectively.

Of the 18 SNPs in group 3, three SNPs were located in the intron of BCL2 associated athanogene 6 (BAG6) gene, two SNPs were located at the ring finger protein 1 (RING1) gene promoter, six SNPs were located at the telomeric end of tripartite motif containing 27 (TRIM27) gene, seven SNPs were located in the exon, intron, or 3′-untranslated region (UTR) of HLA-DOB gene, respectively. None of the SNPs with donor-recipient pair mismatched genotype was related to the risk of relapse (Supplementary Table 3 and 4). On the other hand, the SNP of rs213210 was related the risk of disease relapse for patients with AML as revealed by recipient genotype analysis (genotypic test P = 0.0444, Table 4 and Supplementary Table 5). A greater risk of relapse was associated with the patients who had the polymorphism of G at rs213210 than the patients who had the polymorphism of A at the same SNP position. Donor genotype analysis of these SNPs revealed that rs213210 located at the RING1 promoter was related to the risk of relapse for patients with ALL (genotypic test P = 0.0285; Table 4 and Supplementary Table 6). Donors with G/G genotype of rs213210 resulted in lower risk of relapse for recipients. Additional analysis by Chi-square test revealed that the recipient SNPs of rs17220087 and rs17213693 located in the HLA-DOB intron were related to the risk of relapse for patients with AML (rs17220087: Chi-square test P = 0.0465; rs17213693: Chi-square test P = 0.0465; Table 4) and ALL (rs17220087: Chi-square test P = 0.0311; rs172213693: Chi-square test P = 0.0311; Table 4). One additional recipient SNP of rs2070120 located in the HLA-DOB gene 3′-UTR was related to the risk of relapse for patients with ALL (Chi-square test P = 0.0311; Table 4).

There was no association between the new 7 SNPs identified in this study and the previous 9 SNPs reported by Petersdorf *et al*.^11^ as revealed by the pair-wise linkage disequilibrium (LD) analysis. The newly identified rs213210 and rs17213693 was in high LD with rs107822 (D’ = 0.96) and rs2070120 (D’ = 0.96). In the analysis, D’ was the parameter for normalized standard measurement of LD which compares the observed and expected frequencies of one haplotype comprised by alleles at different loci. This implies that the effects of these SNPs on the risk of post-HSCT relapse might not be independent.

## Discussion

**Figure 1:**
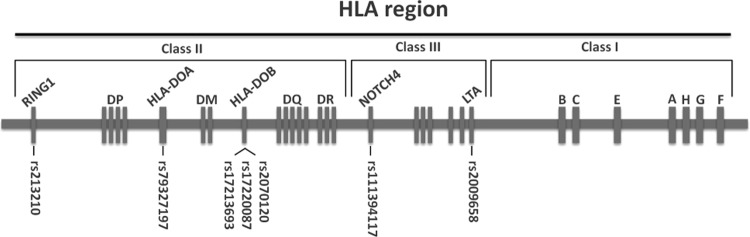
Relative position of the relapse-associated SNPs. Seven relapse-associated SNPs are shown on a map illustrating the HLA region. SNPs are shown by their rs numbers.

By analysis of the DNA from 141 patients with leukemia and their respective HSCT donors, a panel of 7 SNPs in the HLA region was defined to associate with the risk of relapse for patients with leukemia post-HSCT. These SNPs (rs111394117, rs79327197, rs2009658, rs213210, rs17220087, rs17213693, and rs2070120) were located mainly in the NOTCH4, HLA-DOA, HLA-DOB, LTA and RING1 genes (Fig. 1 and Table 4). The disease- and donor/recipient type-specific impacts of these SNPs on the risk of relapse are also demonstrated in this study that can be related to the different mechanistic insight in the pathogenesis of AML and ALL.

**Figure 2:**
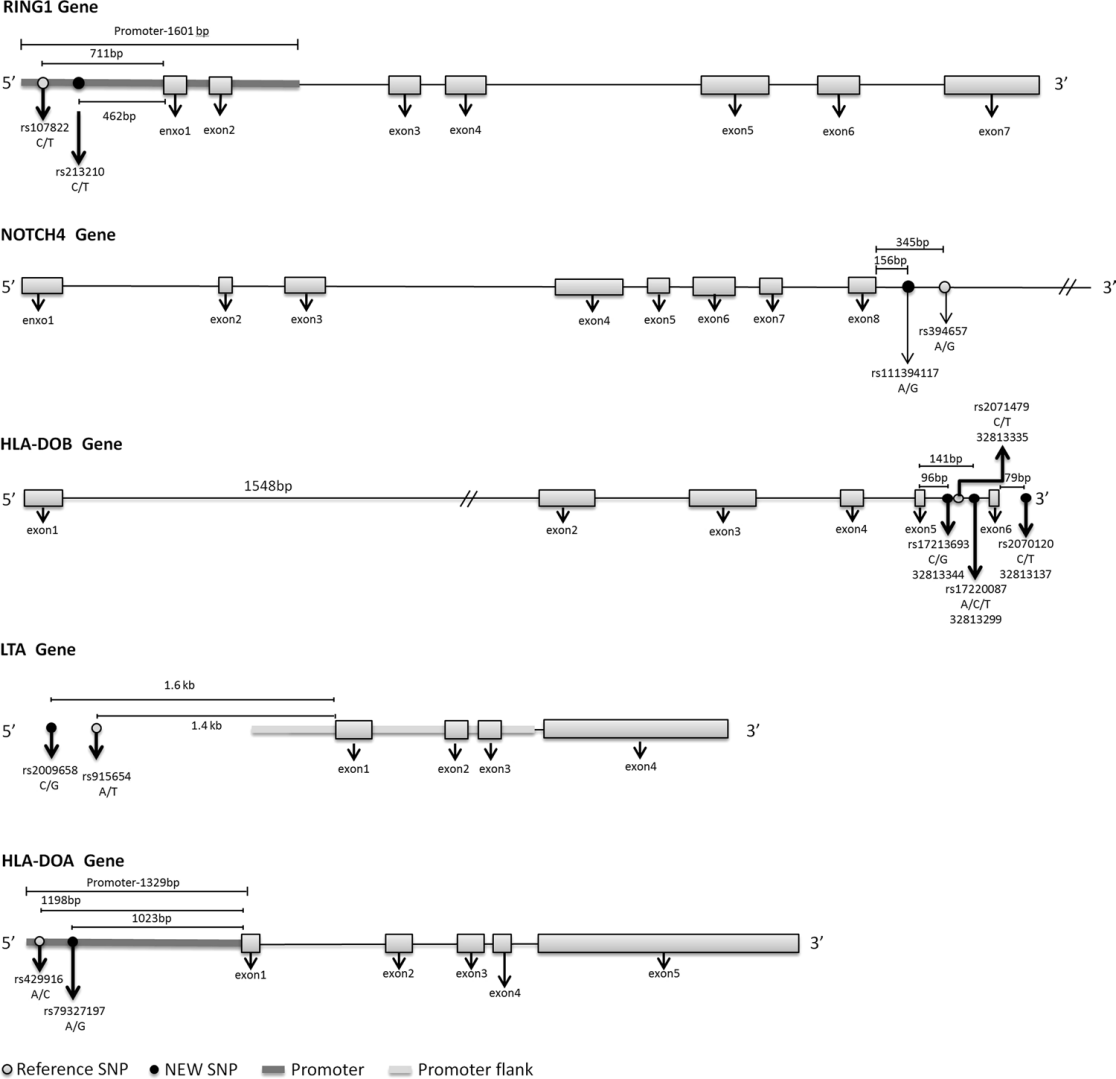
The relative positions of the SNPs that are associated with the risk of post-HSCT relapse. The structures for the genes carrying or nearby the relapse-associated SNPs are shown.

The donor type SNP of rs111394117 is located in the NOTCH4 intron 9, 156 bp from the 3′ of exon 8 (Fig. 2). NOTCH4 encodes a member of the NOTCH family proteins which are involved in differentiation, proliferation and apoptotic programs^15^. Notch signals markedly enhance progenitor expansion and are reported to associate with several types of malignancies such as leukemia and hemangioblastoma^16–18^. Activation of Notch4 enhances stem cell activity, reduces differentiation and alters lymphoid development. Sequence polymorphism of Notch4 receptors could alter the production of cytokines such as TNF-α, IFN-γ, IL-4, and IL-17 that changes the inflammatory status of patients^19^. With the SNP rs111394117 locating in the intron of NOTCH4 gene, alteration of protein structure or function is not likely attributed to the effects of rs111394117 on the relapse for patients with AML. Instead, the polymorphism of G at rs111394117 may cause aberrant splicing and produce protein variants with altered functions of Notch4 leading to a favorable post-HSCT patient status.

The recipient type SNPs of rs79327197, rs17220087, rs17213693, and rs2070120 are located in the HLA-DOA promoter and the HLA-DOB intron or 3′-UTR. All these SNPs attributed to the relapse of patients with ALL, while only the SNPs of rs17220087 and rs17213693 are related to the relapse of patients with AML (Fig. 2). HLA-DO is a heterodimer formed by HLA-DOA and HLA-DOB and plays a role antigen presentation and antigen loading on HLA class II proteins mediated by HLA-DM^20^. Impaired function or down-regulation of HLA-DO leads to less-restrained antigen presentation^21^. The C polymorphism at the SNPs of rs17220087 and the G polymorphism at the SNPs of rs17213693 which are located in HLA-DOB intron are likely to cause aberrant mRNA splicing and produce HLA-DOB protein variants with abnormal activity for accurate antigen presentation. This may cause an increased in relapse for patients with AML and ALL post-HSCT. The SNPs of rs2070120 was located in the 3′-UTR of HLA-DOB gene. With 3′-UTR of RNA transcript usually containing the target sites of regulatory RNA such as miRNA^22,23^, the polymorphism of rs2070120 may be related to the relapse of patients with ALL by modulating the levels of HLA-DOB expression and affecting the normal function of HLA-DO in antigen presentation. On the other hand, the SNP of rs79327197 is located in the HLA-DOA promoter. It is likely that the polymorphism of this SNP causes a decrease in HLA-DOA promoter activity leading to a decrease in HLA-DOA expression, thereby relates to the relapse of patients with ALL.

The recipient type SNP of rs2009658 is related to the risk of relapse for patients with ALL and is located in the telomeric end of LTA gene, 1.6 kb from the 5′-end of exon 1 (Fig. 2). LTA encodes a cytokine produced by lymphocytes^24^ and attributes to the risk of relapse for patients with ALL. The LTA protein induces a variety of inflammation, immune stimulation and anti-viral responses involved in the development of secondary lymphoid organ formation and apoptosis^25^. LTA expression contributes to the development of T-cell acute lymphoblastic leukemia^26^. With the SNP of rs2009658 locating at 200 bp from the 5′-end of the reported promoter region and LTA is an autocrine growth factor for leukemic cells^27^, patients with the polymorphism of G at rs2009658 may enhance the LTA promoter activity by altering the binding affinity or binding pattern of transcription factor(s) to the promoter leading to an increase in LTA expression and contribute to the greater risk of relapse of patients with ALL post-HSCT.

Relapse risk for patients with ALL and AML are associated with the donor genotype and recipient genotype of rs213210, respectively. The SNP is located in the promoter region, 462 bp from exon 1 of RING1 gene (Fig. 2)^28^. RING1 belongs to the PcG family proteins which functions in self-renewal and proliferation of normal cells^29^. The study by Xu’s *et al*. revealed that RING1 is expressed in AML and various subsets of myelodysplastic syndrome. RING1 overexpression drives tumorigenesis and links to poor prognostic scoring for patients with cancer^30^. With rs213210 locating in the promoter region of RING1, patients who carried the polymorphism of G may enhance the RING1 promoter activity by altering the binding affinity or binding pattern of transcription factor(s) to the promoter leading to an increase in RING1 expression. The interaction of RING1 and the oncogenic proteins such as MLL-AF9 and MLL-ENL in the leukemic cells may contribute to the growth of cancer cells and increase the risk of relapse post-HSCT^31,32^.

In this study, the association between SNPs and post-HSCT relapse was analyzed according to the findings by Petersdorf *et al*.^11^. Nevertheless, different SNPs were unveiled as the risk factors for relapse post-HSCT^11^. Difference in ethnicity is a likely explanation for these findings. On the other hand, SNPs beyond the HLA regions have been revealed as the risk factors for relapse post-HSCT. For example, improved survival of HSCT was associated with the SNPs within the tumor necrosis factor II receptor superfamily member 1B (TNFRSF1B) and the interleukin 10 gene^33,34^. Whether or not the risk of relapse post-HSCT is related to the SNPs outside the HLA region remains to be a topic of research interest.

## Conclusions

In regard to the data obtained from this study, seven SNPs located in the HLA region contribute to an increase in the risk of post-HSCT relapse for patients with AML and ALL. The study may have clinical impacts on searching and selecting appropriate donor-recipient pair for HSCT. The genes associated with these SNPs mostly have pathophysiological functions in the immunological disorders. Future investigations are required to demonstrate how these SNPs elucidate their biological effects on the adjacent genes leading to transplantation failure.

## Methods

### Patients and laboratory tests

The Institutional Review Board of Chang Gung Memorial Hospital (CGMH) has reviewed and approved the study. The approval ID was 102-4949B. All methods were performed in accordance with the relevant guidelines and regulations. Patients (n = 141) with AML (n = 85) and ALL (n = 56) receiving HSCT from their donors were recruited at CGMH (Table 1). Written informed consent was provided by all 141 recipients before enrollment in this study.

The sequence-specific oligonucleotide probes-based method, LABType SSO Typing Test (Thermo Fisher, Waltham, MA), was used for HLA typing of HLA-A, -C, -B, -DRB1, and -DQB1 alleles for donors and recipients prior to transplantation. High-resolution HLA typing by the SeCore kit (Thermo Fisher, Waltham, MA) was then performed to obtain more detailed allele information. Allele ambiguity of the SeCore typing was resolved by using sequence-specific primers-based method, the MicroSSP Allele Specific Typing Tray (Thermo Fisher, Waltham, MA).

### Chimerism test and relapse assessment post-HSCT

The AmpFISTR Identifiler amplification kit (Thermo Fisher, Waltham, MA) for analysis of short tandem repeats (STR) was used as the chimerism test to evaluate HSCT engraftment as described previously^35^. Briefly, the following tetranucleotide STR loci were included in the STR analysis: D8S1179, D21S11, D7S820, and CSF1PO (all labeled with 6-FAM blue dye); D3S1358, TH01, D13S317, D16S539, and D2S1338 (all labeled with VIC green dye); and D19S3433, vWA, TPOX, and D18S51 (all labeled with NED yellow dye). Manufacturer’s instruction was followed to set up the cycle conditions of PCR and the analysis of PCR product. Recurrence of malignancy as defined by relapse was based on one or more of the following laboratory findings: reappearance of leukemia blasts in the peripheral blood or >5% blasts in the bone marrow, >0.01% of the peripheral blood cells as analyzed by flow cytometry carrying the individualized minimal residual disease panel for each patient according to the initial CD markers at diagnosis, abnormal karyotypes with the changes for the number of chromosomes or with structural variants of translocation, insertion, and deletion in cytogenetic analysis, and the presence of >5% recipient STR alleles in chimerism test.

### Selection of SNPs

The risk of post-HSCT disease-free survival, patient death, transplant-related death, relapse, and acute and chronic GVHD has been related to the nine SNPs (rs2244546, rs915654, rs429916, rs2242656, rs209130, rs2075800, rs394657, rs2071479, rs107822) within the HLA region in a previous study^11^. These SNPs were considered as the sourced SNPs in this study and were classified into three groups including donor genotype, recipient genotype, and mismatched donor-recipient pair genotype (Table 2). The classification was determined according to whether donor or recipient SNP or mismatch of donor-recipient pair SNP conferred the relapse-associated risks.

### PCR and sequencing

The PCR and sequencing was performed as described previously^35^. Briefly, the QIAamp DNA Blood mini Kit (Qiagen, Valencia, CA) was used to extract the genomic DNA from 3 ml peripheral blood. The DNA fragments that flanking 500 bps of the 9 sourced SNPs were amplified by using 9 different primer pairs (Table 3), respectively. In a reaction volume of 50 μl containing 1X reaction buffer, 10 nmol of dNTP, 6 pmol of forward and reversed primers, 300 ng of genomic DNA, and 1 μl of *Pfu Turbo* Hotstart DNA Polymerase (Agilent, Santa Clara, CA), PCR was performed with the following cycling condition: 4 min at 94 °C for 1 cycle, 30 sec at 94 °C, 30 sec at 58 °C, and 45 sec at 72 °C for 30 cycles, and 10 min at 72 °C for 1 cycle. When PCR was completed, 5 μl of PCR products were fractionated on a 2% agarose gel and analyzed by ethidium bromide staining. The Big Dye Terminator Cycle Sequencing kit (Thermo Fisher, Waltham, MA) and an ABI PRISM Genetic Analyzer (Thermo Fisher, Waltham, MA) were used for direct sequencing of the remaining PCR product based on the instruction of the manufacturer. SNPs data were not available for all donor-recipient pairs because of insufficient amount of genomic DNA and failure of PCR reaction.

### Statistical analysis

The analysis was performed as previously described^35^. Briefly, the quality of SNPs testing experiments was examined by using the Hardy-Weinberg equilibrium (HWE) test. Those SNPs which infringed on the HWE test were excluded from the analysis. The association of disease relapse with candidate SNPs was evaluated by calculating and comparing the allele and genotype frequencies between the non-relapse and relapse groups. Whether the specific SNP genotypes related to the risk of post-HSCT relapse was evaluated by a genotypic test. Chi-square test was further used to search for additional SNP genotypes that were related to the risk of relapse. For the mode of donor-recipient pair analysis, the association for the risk of disease relapse with the mismatch status of SNP genotypes was evaluated by the chi-square and Fisher’s exact tests. HaploView 4.2 software (https://www.broadinstitute.org/haploview/haploview) was used for pair-wise LD analysis of SNPs^36^.

## Supplementary information

Supplementary Table

## Data Availability

The raw data and statistical data used in this study were included in the Supplemental Files.
