# Supplementary material for: Association between single nucleotide polymorphisms within HLA region and disease relapse for patients with hematopoietic stem cell transplantation
Source: Sci Rep. 2019 Sep 24;9:13731. doi: 10.1038/s41598-019-50111-5 (PMC6760494; doi:10.1038/s41598-019-50111-5)
Supplement: Supplementary file 1 — Supplementary Table [file 41598_2019_50111_MOESM1_ESM.pdf]

Supplementary Information

Association between single nucleotide polymorphisms within HLA region and disease relapse for patients with hematopoietic stem cell transplantation

Ding-Ping Chen, Su-Wei Chang, Po-Nan Wang, Fang-Ping Hus, Ching-Ping Tseng

Supplementary Table 1. The association of group 1 SNPs with the risk of relapse for patients with HSCT.

SNP	Physical position[¥] (bp)	Gene	Source SNP [§]	Disease/ Status	Risk allele frequency Number of allele (%)	Donor genotype frequency Number of donor (%)			*P*
rs2523675	31468255	2.4 kb telomeric of HCP5	rs2244546	AML	A	A/A	A/G	G/G	0.4578
				Relapse	22 (42.3)	5 (19.2)	12 (46.2)	9 (34.6)	
				Non-relapse	41 (35.3)	11 (19.0)	19 (32.8)	28 (48.3)	
				ALL	G	A/A	A/G	G/G	0.4792
				Relapse	17 (56.7)	3 (20.0)	7 (46.7)	5 (33.3)	
				Non-relapse	38 (46.3)	15 (36.6)	14 (34.2)	12 (29.3)	
rs2518028	31468270	2.5 kb telomeric of HCP5	rs2244546	AML	T	C/C	C/T	T/T	0.1632
				Relapse	14 (26.9)	16 (61.5)	6 (23.1)	4 (15.4)	
				Non-relapse	21 (18.1)	39 (67.2)	17 (29.3)	2 (3.5)	
				ALL	C	C/C	C/T	T/T	1
				Relapse	25 (83.3)	11 (73.3)	3 (20.0)	1 (6.7)	
				Non-relapse	68 (82.9)	30 (73.2)	8 (19.5)	3 (7.3)	
rs141431529	31468278	2.5 kb telomeric of HCP5	rs2244546	AML	G	G/G	G/T	T/T	0.7477
				Relapse	49 (94.2)	23 (88.5)	3 (11.5)	0 (0.0)	
				Non-relapse	107 (92.2)	49 (84.5)	9 (15.5)	0 (0.0)	
				ALL	T	G/G	G/T	T/T	0.5616
				Relapse	2 (6.7)	13 (86.7)	2 (13.3)	0 (0.0)	
				Non-relapse	2 (2.4)	39 (95.1)	2 (4.9)	0 (0.0)	
rs2256594	32219095	NOTCH4, intron	rs394657	AML	A	A/A	A/G	G/G	1
				Relapse	35 (67.3)	12 (46.2)	11 (42.3)	3 (11.5)	
				Non-relapse	73 (65.2)	24 (42.9)	25 (44.6)	7 (12.5)	
				ALL	G	A/A	A/G	G/G	0.217
				Relapse	12 (40.0)	5 (33.3)	8 (53.3)	2 (13.3)	
				Non-relapse	31 (38.8)	19 (47.5)	11 (27.5)	10 (25.0)	
rs111394117	32219436	NOTCH4, intron	rs394657	AML	A	A/A	A/G	G/G	0.5197
				Relapse	2 (4.0)	1 (4.0)	0 (0.0)	24 (96.0)	
				Non-relapse	1 (0.9)	0 (0.0)	1 (1.8)	56 (98.3)	
				ALL	A	A/A	A/G	G/G	0.0166
				Relapse	4 (13.3)	1 (6.7)	2 (13.3)	12 (80.0)	
				Non-relapse	0 (0.0)	0 (0.0)	0 (0.0)	40 (100.0)	

[¥]Assembly version: GRCh37.p13. [§]The sequenced SNPs were selected and studied based on the transplant determinants identified by Petersdorf *et al.* (2013).

Supplementary Table 2. The association of group 2 SNPs with the risk of relapse for patients with HSCT.

SNP	Physical position[¥] (bp)	Gene	Source SNP[§]	Disease/ Status	Risk allele frequency Number of allele (%)	Recipient's genotype frequency Number of patients (%)			*P*
rs9276982	33010438	HLA-DOA, promoter	rs429916	AML	G	A/A	A/G	G/G	0.3076
				Relapse	39 (88.6)	0 (0.0)	5 (22.7)	17 (77.3)	
				Non-relapse	87 (77.7)	1 (1.8)	23 (41.1)	32 (57.1)	
				ALL	A	A/A	A/G	G/G	1
				Relapse	7 (23.3)	1 (6.7)	5 (33.3)	9 (60.0)	
				Non-relapse	15 (20.3)	1 (2.7)	13 (35.1)	23 (62.2)	
rs71565361	33010551	HLA-DOA, promoter	rs429916	AML	A	A/A	A/C	C/C	0.2752
				Relapse	1 (2.3)	0 (0.0)	1 (4.6)	21 (95.5)	
				Non-relapse	0 (0.0)	0 (0.0)	0 (0.0)	56 (100.0)	
				ALL	C	A/A	A/C	C/C	N/A
				Relapse	30 (100.0)	0 (0.0)	0 (0.0)	15 (100.0)	
				Non-relapse	74 (100.0)	0 (0.0)	0 (0.0)	37 (100.0)	
rs79327197	33010635	HLA-DOA, promoter	rs429916	AML	A	A/A	A/G	G/G	1
				Relapse	40 (90.9)	18 (81.8)	4 (18.2)	0 (0.0)	
				Non-relapse	101 (90.2)	45 (80.4)	11 (19.6)	0 (0.0)	
				ALL	G	A/A	A/G	G/G	0.015
				Relapse	5 (16.7)	10 (66.7)	5 (33.3)	0 (0.0)	
				Non-relapse	2 (2.7)	35 (94.6)	2 (5.4)	0 (0.0)	
rs151190962	33010881	HLA-DOA, promoter	rs429916	AML	-	A/A	A/-	-/-	0.6087
				Relapse	2 (4.6)	20 (90.9)	2 (9.1)	0 (0.0)	
				Non-relapse	3 (2.7)	53 (94.6)	3 (5.4)	0 (0.0)	
				ALL	-	A/A	A/-	-/-	0.2858
				Relapse	1 (3.3)	14 (93.3)	1 (6.7)	0 (0.0)	
				Non-relapse	0 (0.0)	37 (100.0)	0 (0.0)	0 (0.0)	
rs9282369	33011011-12	1.4 kb centromeric of HLA-DOA	rs429916	AML	T	T/T	T/-	-/-	0.6105
				Relapse	27 (61.4)	8 (36.4)	11 (50.0)	3 (13.6)	
				Non-relapse	59 (52.7)	17 (30.4)	25 (44.6)	14 (25.0)	
				ALL	T	T/T	T/-	-/-	0.7606
				Relapse	13 (43.3)	3 (20.0)	7 (46.7)	5 (33.3)	
				Non-relapse	31 (41.9)	5 (13.5)	21 (56.8)	11 (29.7)	

rs2009658	31538244	1.6 kb telomeric of LTA	rs915654	AML	G	C/C	C/G	G/G	0.4124
				Relapse	6 (14.3)	16 (76.2)	4 (19.1)	1 (4.8)	
				Non-relapse	9 (8.5)	44 (83.0)	9 (17.0)	0 (0.0)	
				ALL	G	C/C	C/G	G/G	0.0148
				Relapse	5 (16.7)	12 (80.0)	1 (6.7)	2 (13.3)	
				Non-relapse	12 (16.2)	25 (67.6)	12 (32.4)	0 (0.0)	
rs736160	31538496-97	1.4 kb telomeric of LTA	rs915654	AML	T	A/A	A/T	T/T	1
				Relapse	40 (100.0)	0 (0.0)	0 (0.0)	20 (100.0)	
				Non-relapse	105 (99.1)	0 (0.0)	1 (1.9)	52 (98.1)	
				ALL	T	A/A	A/T	T/T	N/A
				Relapse	30 (100.0)	0 (0.0)	0 (0.0)	15 (100.0)	
				Non-relapse	74 (100.0)	0 (0.0)	0 (0.0)	37 (100.0)	
rs915654	31538497	1.4 kb telomeric of LTA	rs915654	AML	A	A/A	A/T	T/T	0.2598
				Relapse	26 (65.0)	9 (45.0)	8 (40.0)	3 (15.0)	
				Non-relapse	54 (50.9)	13 (24.5)	28 (52.8)	12 (22.6)	
				ALL	T	A/A	A/T	T/T	0.1895
				Relapse	16 (53.3)	4 (26.7)	6 (40.0)	5 (33.3)	
				Non-relapse	27 (36.5)	14 (37.8)	19 (51.4)	4 (10.8)	
rs371621895	31809997	HSPA1L, exon	rs2075800	AML	G	G/G	G/-	-/-	0.8057
				Relapse	40 (87.0)	18 (78.3)	4 (17.4)	1 (4.4)	
				Non-relapse	98 (84.5)	42 (72.4)	14 (24.1)	2 (3.5)	
				ALL	G	G/G	G/-	-/-	0.1235
				Relapse	30 (100.0)	15 (100.0)	0 (0.0)	0 (0.0)	
				Non-relapse	69 (86.3)	30 (75.0)	9 (22.5)	1 (2.5)	
rs2075800	31810169	HSPA1L, exon	rs2075800	AML	T	C/C	C/T	T/T	0.2553
				Relapse	18 (40.9)	7 (31.8)	12 (54.6)	3 (13.6)	
				Non-relapse	43 (38.4)	25 (44.6)	19 (33.9)	12 (21.4)	
				ALL	C	C/C	C/T	T/T	0.6245
				Relapse	20 (66.7)	7 (46.7)	6 (40.0)	2 (13.3)	
				Non-relapse	43 (55.1)	13 (33.3)	17 (43.6)	9 (23.1)	
rs2227956	31810495	HSPA1L, exon	rs2075800	AML	A	A/A	A/G	G/G	0.460
				Relapse	39 (88.6)	17 (77.3)	5 (22.7)	0 (0.0)	
				Non-relapse	91 (81.3)	40 (71.4)	11 (19.6)	5 (8.9)	

ALL	A	A/A	A/G	G/G	0.665
Relapse	25 (83.3)	10 (66.7)	5 (33.3)	0 (0.0)	
Non-relapse	64 (82.1)	27 (69.2)	10 (25.6)	2 (5.1)	

[¥]Assembly version: GRCh37.p13. [§]The sequenced SNPs were selected and studied based on the transplant determinants identified by Petersdorf et al. (2013).

Supplementary Table 3. The association of group 3 SNPs with the risk of relapse for AML patients with HSCT as analyzed by Chi-square test and Fisher's exact test.

SNP	Physical position[¥] (bp)	Gene	Source[§]	Genotypes of donor-recipient pairs Number of patients (%)		Chi-square test P	Fisher's exact test P
				Matched	Not matched		
rs3130048	31645962	BAG6, intron	rs2242656			0.9620	1.0000
Relapse				18 (81.8)	4 (18.2)		
Non-relapse				48 (81.4)	11 (18.6)		
rs2844464	31646214	BAG6, intron	rs2242656			0.4719	0.7158
Relapse				23 (92.0)	2 (8.0)		
Non-relapse				51 (86.4)	8 (13.6)		
rs2242656	31646325	BAG6, intron	rs2242656			0.4719	0.7158
Relapse				23 (92.0)	2 (8.0)		
Non-relapse				51 (86.4)	8 (13.6)		
rs107822	33207798	RING, promoter	rs107822			0.0729	0.0812
Relapse				19 (79.2)	5 (20.8)		
Non-relapse				32 (58.2)	23 (41.8)		
rs213210	33208047	RING, promoter	rs107822			0.1999	0.3063
Relapse				18 (75.0)	6 (25.0)		
Non-relapse				33 (60.0)	22 (40.0)		
rs209132	28899705	3.3 kb telomeric of TRIM27	rs209130			0.1657	0.3026
Relapse				11 (84.6)	2 (15.4)		
Non-relapse				27 (64.3)	15 (35.7)		
rs209131	28899978	3 kb telomeric of TRIM27	rs209130			0.3754	0.5128
Relapse				10 (76.9)	3 (23.1)		
Non-relapse				30 (63.8)	17 (36.2)		
rs209130	28900023	3 kb telomeric of	rs209130			0.1209	0.1859

Relapse		TRIM27		11 (84.6)	2 (15.4)		
Non-relapse				29 (61.7)	18 (38.3)		
rs1536215	28900138	2.9 kb telomeric of TRIM27	rs209130			0.8891	1.0000
Relapse				10 (76.9)	3 (23.1)		
Non-relapse				37 (78.7)	10 (21.3)		
rs139791445	28900314	2.7 kb telomeric of TRIM27	rs209130			0.4284	1.0000
Relapse				13 (100.0)	0 (0.0)		
Non-relapse				41 (95.4)	2 (4.7)		
rs6928948	28900440	2.6 kb telomeric of TRIM27	rs209130			0.3302	0.6478
Relapse				8 (88.9)	1 (11.1)		
Non-relapse				19 (73.1)	7 (26.9)		
rs11244	32812947	HLA-DOB, 3'UTR[$]	rs2071479			0.4728	0.5823
Relapse				17 (77.3)	5 (22.7)		
Non-relapse				38 (69.1)	17 (30.9)		
rs2070120	32813137	HLA-DOB, 3'UTR	rs2071479			0.6256	0.6909
Relapse				21 (87.5)	3 (12.5)		
Non-relapse				51 (91.1)	5 (8.9)		
rs41258084	32813180	HLA-DOB, 3'UTR	rs2071479			0.4521	0.4288
Relapse				21 (87.5)	3 (12.5)		
Non-relapse				51 (92.7)	4 (7.27)		
rs17220087	32813299	HLA-DOB, intron	rs2071479			0.8949	1.0000
Relapse				22 (91.7)	2 (8.3)		
Non-relapse				49 (90.7)	5 (9.3)		
rs2071479	32813335	HLA-DOB, intron	rs2071479			0.4677	0.6696
Relapse				21 (87.5)	3 (12.5)		
Non-relapse				50 (92.6)	4 (7.4)		
rs17213693	32813344	HLA-DOB, intron	rs2071479			0.8949	1.0000

Relapse				22 (91.7)	2 (8.3)		
Non-relapse				49 (90.7)	5 (9.3)		
rs2070121	32813777	HLA-DOB, exon	rs2071479			N/A[#]	N/A
Relapse				20 (100.0)	0 (0.0)		
Non-relapse				41 (100.0)	0 (0.0)		

[¥]Assembly version: GRCh37.p13. [$]The sequenced SNPs were selected and studied based on the transplant determinants identified by Petersdorf et al. (2013).
[$]UTR: untranslated region. [#]N/A: not available.

Supplementary Table 4. The association of group 3 SNPs with the risk of relapse for ALL patients with HSCT as analyzed by Chi-square test and Fisher's exact test.

SNP	Physical position[¥] (bp)	Gene	Source[§]	Genotypes of donor-recipient pairs Number of patients (%)		Chi-square test P	Fisher's exact test P
				Matched	Not matched		
rs3130048	31645962	BAG6, intron	rs2242656			0.4677	0.6639
Relapse				13 (92.9)	1 (7.1)		
Non-relapse				35 (85.4)	6 (14.6)		
rs2844464	31646214	BAG6, intron	rs2242656			0.5536	1.0000
Relapse				14 (93.3)	1 (6.7)		
Non-relapse				36 (87.8)	5 (12.2)		
rs2242656	31646325	BAG6, intron	rs2242656			0.2034	0.5655
Relapse				15 (100.0)	0 (0.0)		
Non-relapse				36 (90.0)	4 (10.0)		
rs107822	33207798	RING, promoter	rs107822			0.2419	0.4162
Relapse				10 (90.9)	1 (9.1)		
Non-relapse				29 (74.4)	10 (25.6)		
rs213210	33208047	RING, promoter	rs107822			0.8232	1.0000
Relapse				8 (72.7)	3 (27.3)		
Non-relapse				27 (69.2)	12 (30.8)		
rs209132	28899705	3.3 kb telomeric of TRIM27	rs209130			0.2574	0.4077
Relapse				8 (66.7)	4 (33.3)		
Non-relapse				24 (82.8)	5 (17.2)		
rs209131	28899978	3 kb telomeric of TRIM27	rs209130			0.6216	0.7108
Relapse				8 (66.7)	4 (33.3)		
Non-relapse				23 (74.2)	8 (25.8)		
rs209130	28900023	3 kb telomeric of	rs209130			0.6421	0.7272

Relapse		TRIM27		9 (75.0)	3 (25.0)		
Non-relapse				21 (67.7)	10 (32.3)		
rs1536215	28900138	2.9 kb telomeric of TRIM27	rs209130			0.8390	1.0000
Relapse				10 (83.3)	2 (16.7)		
Non-relapse				25 (80.7)	6 (19.4)		
rs139791445	28900314	2.7 kb telomeric of TRIM27	rs209130			0.4918	0.4948
Relapse				11 (91.7)	1 (8.3)		
Non-relapse				29 (96.7)	1 (3.3)		
rs6928948	28900440	2.6 kb telomeric of TRIM27	rs209130			0.9200	1.0000
Relapse				6 (75.0)	2 (25.0)		
Non-relapse				10 (76.9)	3 (23.1)		
rs11244	32812947	HLA-DOB, 3'UTR[$]	rs2071479			0.8939	1.0000
Relapse				12 (85.7)	2 (14.3)		
Non-relapse				32 (84.2)	6 (15.8)		
rs2070120	32813137	HLA-DOB, 3'UTR	rs2071479			0.9158	1.0000
Relapse				12 (85.7)	2 (14.3)		
Non-relapse				33 (86.8)	5 (13.2)		
rs41258084	32813180	HLA-DOB, 3'UTR	rs2071479			0.1139	0.1741
Relapse				14 (100.0)	0 (0.0)		
Non-relapse				32 (84.2)	6 (15.8)		
rs17220087	32813299	HLA-DOB, intron	rs2071479			0.8939	1.0000
Relapse				12 (85.7)	2 (14.3)		
Non-relapse				32 (84.2)	6 (15.8)		
rs2071479	32813335	HLA-DOB, intron	rs2071479			N/A[#]	N/A
Relapse				14 (100.0)	0 (0.0)		
Non-relapse				38 (100.0)	0 (0.0)		
rs17213693	32813344	HLA-DOB, intron	rs2071479			0.9158	1.0000

Relapse				12 (85.7)	2 (14.3)		
Non-relapse				33 (86.8)	5(13.2)		
rs2070121	32813777	HLA-DOB, exon	rs2071479			N/A	N/A
Relapse				11 (100.0)	0 (0.0)		
Non-relapse				28 (100.0)	0 (0.0)		

[¥]Assembly version: GRCh37.p13. [§]The sequenced SNPs were selected and studied based on the transplant determinants identified by Petersdorf et al. (2013).
[$]UTR: untranslated region. [#]N/A: not available.

Supplementary Table 5. The association of group 3 SNPs with the risk of relapse for AML patients with HSCT as analyzed by genotypic test.

**SNP**	**Physical Position[¥] (bp)**	**Gene**	**Source[§]**	**Donor's genotype frequency Number of patients (%)**			**P**	**Recipient's genotype frequency Number of patients (%)**			**P**
rs3130048	31645962	BAG6, intron	rs2242656	C/C	C/T	T/T	0.1315	C/C	C/T	T/T	0.0856
Relapse				0 (0.0)	12 (50.0)	12 (50.0)		0 (0.0)	12 (54.6)	10 (45.5)	
Non-relapse				7 (11.9)	20 (33.9)	32 (54.2)		8 (13.6)	19 (32.2)	32 (54.2)	
rs2844464	31646214	BAG6, intron	rs2242656	A/A	A/G	G/G	1.0000	A/A	A/G	G/G	1.0000
Relapse				1 (4.0)	7 (28.0)	17 (68.0)		1 (4.0)	7 (28.0)	17 (68.0)	
Non-relapse				4 (6.8)	17 (28.8)	38 (64.4)		4 (6.8)	17 (28.8)	38 (64.4)	
rs2242656	31646325	BAG6, intron	rs2242656	C/C	C/T	T/T	1.0000	C/C	C/T	T/T	1.0000
Relapse				1 (4.0)	7 (28.0)	17 (68.0)		1 (4.0)	7 (28.0)	17 (68.0)	
Non-relapse				4 (6.8)	17 (28.8)	38 (64.4)		4 (6.8)	17 (28.8)	38 (64.4)	
rs107822	33207798	RING, promoter	rs107822	C/C	C/T	T/T	0.5097	C/C	C/T	T/T	0.3171
Relapse				2 (7.7)	15 (57.7)	9 (34.6)		1 (4.2)	12 (50.0)	11 (45.8)	
Non-relapse				9 (16.4)	26 (47.3)	20 (36.4)		9 (15.5)	30 (51.7)	19 (32.8)	
rs213210	33208047	RING, promoter	rs107822	A/A	A/G	G/G	0.1164	A/A	A/G	G/G	0.0444
Relapse				3 (11.5)	17 (65.4)	6 (23.1)		2 (8.3)	13 (54.2)	9 (37.5)	
Non-relapse				16 (29.1)	23 (41.8)	16 (29.1)		20 (34.5)	26 (44.8)	12 (20.7)	
rs209132	28899705	3.3 kb telomeric of TRIM27	rs209130	A/A	A/G	G/G	0.6625	A/A	A/G	G/G	0.4764
Relapse				1 (6.3)	7 (43.8)	8 (50.0)		1 (6.3)	8 (50.0)	7 (43.8)	
Non-relapse				3 (7.0)	12 (27.9)	28 (65.1)		5 (9.3)	17 (31.5)	32 (59.3)	
rs209131	28899978	3 kb telomeric of TRIM27	rs209130	A/A	A/G	G/G	0.5660	A/A	A/G	G/G	1.0000
Relapse				3 (18.8)	10 (62.5)	3 (18.8)		3 (18.8)	8 (50.0)	5 (31.3)	
Non-relapse				11 (23.4)	22 (46.8)	14 (29.8)		10 (18.5)	27 (50.0)	17 (31.5)	
rs209130	28900023	3 kb telomeric of TRIM27	rs209130	C/C	C/T	T/T	1.0000	C/C	C/T	T/T	0.9116
Relapse				1 (6.3)	8 (50.0)	7 (43.8)		1 (6.3)	9 (56.3)	6 (37.5)	
Non-relapse				3 (6.4)	23 (48.9)	21 (44.7)		5 (9.3)	26 (48.2)	23 (42.6)	

rs1536215	28900138	2.9 kb telomeric of TRIM27	rs209130	C/C	C/G	G/G	0.8924	C/C	C/G	G/G	0.5405
Relapse				11 (68.8)	4 (25.0)	1 (6.3)		13 (81.3)	3 (18.8)	0 (0.0)	
Non-relapse				29 (61.7)	16 (34.0)	2 (4.3)		39 (72.2)	15 (27.8)	0 (0.0)	
rs139791445	28900314	2.7 kb telomeric of TRIM27	rs209130	C/C	C/G	G/G	0.6088	C/C	C/G	G/G	1.0000
Relapse				16 (100.0)	0 (0.0)	0 (0.0)		16 (100.0)	0 (0.0)	0 (0.0)	
Non-relapse				45 (95.7)	2 (4.3)	0 (0.0)		49 (96.1)	2 (3.9)	0 (0.0)	
rs6928948	28900440	2.6 kb telomeric of TRIM27	rs209130	A/A	A/C	C/C	0.6968	A/A	A/C	C/C	1.0000
Relapse				0 (0.0)	2 (15.4)	11 (84.6)		0 (0.0)	2 (20.0)	8 (80.0)	
Non-relapse				0 (0.0)	8 (23.5)	26 (76.5)		0 (0.0)	6 (15.8)	32 (84.2)	
rs11244	32812947	HLA-DOB, 3'-UTR[$]	rs2071479	A/A	A/G	G/G	0.6336	A/A	A/G	G/G	0.8695
Relapse				0 (0.0)	11 (45.8)	13 (54.2)		1 (4.2)	10 (41.7)	13 (54.2)	
Non-relapse				0 (0.0)	22 (39.3)	34 (60.7)		5 (8.8)	22 (38.6)	30 (52.6)	
rs2070120	32813137	HLA-DOB, 3'-UTR	rs2071479	A/A	A/G	G/G	1.0000	A/A	A/G	G/G	0.0916
Relapse				0 (0.0)	3 (12.5)	21 (87.5)		0 (0.0)	0 (0.0)	26 (100.0)	
Non-relapse				0 (0.0)	7 (12.5)	49 (87.5)		0 (0.0)	7 (12.1)	51 (87.9)	
rs41258084	32813180	HLA-DOB, 3'-UTR	rs2071479	C/C	C/T	T/T	0.5952	C/C	C/T	T/T	1.0000
Relapse				23 (95.8)	1 (4.2)	0 (0.0)		23 (92.0)	2 (8.0)	0 (0.0)	
Non-relapse				48 (85.7)	7 (12.5)	1 (1.8)		50 (87.7)	6 (10.5)	1 (1.8)	
rs17220087	32813299	HLA-DOB, intron	rs2071479	A/A	A/C	C/C	1.0000	A/A	A/C	C/C	0.0999
Relapse				0 (0.0)	2 (8.3)	22 (91.7)		0 (0.0)	0 (0.0)	25 (100.0)	
Non-relapse				0 (0.0)	6 (10.7)	50 (89.3)		0 (0.0)	8 (14.3)	48 (85.7)	
rs2071479	32813335	HLA-DOB, intron	rs2071479	C/C	C/T	T/T	1.0000	C/C	C/T	T/T	1.0000
Relapse				22 (91.7)	2 (8.3)	0 (0.0)		23 (92.0)	1 (4.0)	1 (4.0)	
Non-relapse				52 (92.9)	4 (7.1)	0 (0.0)		53 (94.6)	2 (3.6)	1 (1.8)	
rs17213693	32813344	HLA-DOB, intron	rs2071479	C/C	C/G	G/G	1.0000	C/C	C/G	G/G	0.0996
Relapse				0 (0.0)	2 (8.3)	22 (91.7)		0 (0.0)	0 (0.0)	25 (100.0)	
Non-relapse				1 (1.8)	6 (10.7)	49 (87.5)		0 (0.0)	8 (14.3)	48 (85.7)	

rs2070121	32813777	HLA-DOB, exon	rs2071479	C/C	C/G	G/G	N/A[#]	C/C	C/G	G/G	N/A
Relapse				0 (0.0)	0 (0.0)	22 (100.0)		0 (0.0)	0 (0.0)	21 (100.0)	
Non-relapse				0 (0.0)	0 (0.0)	45 (100.0)		0 (0.0)	0 (0.0)	42 (100.0)	

[¥]Assembly version: GRCh37.p13. [§]The sequenced SNPs were selected and studied based on the transplant determinants identified by Petersdorf et al. (2013).
[$]UTR: untranslated region. [#]N/A: not available.

Supplementary Table 6. The association of group 3 SNPs with the risk of relapse for ALL patients with HSCT as analyzed by genotypic test.

SNP	Physical Position[¥] (bp)	Gene/	Source[§]	Donor's genotype frequency Number of patients (%)			P	Recipient's genotype frequency Number of patients (%)			P
rs3130048	31645962	BAG6, intron	rs2242656	C/C	C/T	T/T	0.6585	C/C	C/T	T/T	0.5233
Relapse				2 (13.3)	6 (40.0)	7 (46.7)		3 (21.4)	6 (42.9)	5 (35.7)	
Non-relapse				2 (4.9)	18 (43.9)	21 (51.2)		5 (12.2)	14 (34.2)	22 (53.7)	
rs2844464	31646214	BAG6, intron	rs2242656	A/A	A/G	G/G	0.5745	A/A	A/G	G/G	0.4628
Relapse				0 (0.0)	4 (26.7)	11 (73.3)		0 (0.0)	3 (20.0)	12 (80.0)	
Non-relapse				4 (9.8)	11 (26.8)	26 (63.4)		2 (4.9)	13 (31.7)	26 (63.4)	
rs2242656	31646325	BAG6, intron	rs2242656	C/C	C/T	T/T	0.4279	C/C	C/T	T/T	0.4677
Relapse				0 (0.0)	3 (20.0)	12 (80.0)		0 (0.0)	3 (20.0)	12 (80.0)	
Non-relapse				4 (10.0)	10 (25.0)	26 (65.0)		2 (4.9)	13 (31.7)	26 (63.4)	
rs107822	33207798	RING, promoter	rs107822	C/C	C/T	T/T	0.3569	C/C	C/T	T/T	0.7295
Relapse				2 (16.7)	7 (58.3)	3 (25.0)		2 (14.3)	7 (50.0)	5 (35.7)	
Non-relapse				5 (12.5)	15 (37.5)	20 (50.0)		3 (7.7)	18 (46.2)	18 (46.2)	
rs213210	33208047	RING, promoter	rs107822	A/A	A/G	G/G	0.0285	A/A	A/G	G/G	0.4223
Relapse				3 (25.0)	8 (66.7)	1 (8.3)		4 (28.6)	7 (50.0)	3 (21.4)	
Non-relapse				10 (25.0)	12 (30.0)	18 (45.0)		6 (15.4)	18 (46.2)	15 (38.5)	
rs209132	28899705	3.3 kb telomeric of TRIM27	rs209130	A/A	A/G	G/G	0.4215	A/A	A/G	G/G	0.3554
Relapse				1 (7.7)	2 (15.4)	10 (76.9)		0 (0.0)	3 (23.1)	10 (76.9)	
Non-relapse				3 (8.3)	13 (36.1)	20 (55.6)		3 (9.7)	11 (35.5)	17 (54.8)	
rs209131	28899978	3 kb telomeric of TRIM27	rs209130	A/A	A/G	G/G	0.3693	A/A	A/G	G/G	1.0000
Relapse				1 (7.7)	9 (69.2)	3 (23.1)		1 (7.7)	7 (53.9)	5 (38.5)	
Non-relapse				7 (19.4)	16 (44.4)	13 (36.1)		3 (9.1)	18 (54.6)	12 (36.4)	
rs209130	28900023	3 kb telomeric of TRIM27	rs209130	C/C	C/T	T/T	0.3241	C/C	C/T	T/T	0.7649
Relapse				1 (7.7)	3 (23.1)	9 (69.2)		1 (7.7)	4 (30.8)	8 (61.5)	

Non-relapse				2 (5.6)	18 (50.0)	16 (44.4)		2 (6.1)	15 (45.5)	16 (48.5)	
rs1536215	28900138	2.9 kb telomeric of TRIM27	rs209130	C/C	C/G	G/G	0.5266	C/C	C/G	G/G	1.0000
Relapse				7 (53.9)	5 (38.5)	1 (7.7)		9 (69.2)	4 (30.8)	0 (0.0)	
Non-relapse				25 (69.4)	10 (27.8)	1 (2.8)		24 (72.7)	9 (27.3)	0 (0.0)	
rs139791445	28900314	2.7 kb telomeric of TRIM27	rs209130	C/C	C/G	G/G	0.1797	C/C	C/G	G/G	0.5839
Relapse				11 (84.6)	2 (15.4)	0 (0.0)		13 (100.0)	0 (0.0)	0 (0.0)	
Non-relapse				34 (97.1)	1 (2.9)	0 (0.0)		30 (93.8)	2 (6.3)	0 (0.0)	
rs6928948	28900440	2.6 kb telomeric of TRIM27	rs209130	A/A	A/C	C/C	0.4398	A/A	A/C	C/C	1.0000
Relapse				1 (10.0)	1 (10.0)	8 (80.0)		0 (0.0)	0 (0.0)	9 (100.0)	
Non-relapse				0 (0.0)	2 (8.3)	22 (91.7)		0 (0.0)	1 (6.3)	15 (93.8)	
rs11244	32812947	HLA-DOB, 3'-UTR$	rs2071479	A/A	A/G	G/G	1.0000	A/A	A/G	G/G	0.8942
Relapse				1 (6.7)	4 (26.7)	10 (66.7)		0 (0.0)	4 (28.6)	10 (71.4)	
Non-relapse				3 (7.7)	10 (25.6)	26 (66.7)		2 (5.3)	12 (31.6)	24 (63.2)	
rs2070120	32813137	HLA-DOB, 3'-UTR	rs2071479	A/A	A/G	G/G	1.0000	A/A	A/G	G/G	0.0642
Relapse				0 (0.0)	3 (20.0)	12 (80.0)		0 (0.0)	3 (20.0)	12 (80.0)	
Non-relapse				0 (0.0)	6 (15.4)	33 (84.6)		0 (0.0)	1 (2.6)	37 (97.4)	
rs41258084	32813180	HLA-DOB, 3'-UTR	rs2071479	C/C	C/T	T/T	0.4159	C/C	C/T	T/T	0.6662
Relapse				0 (0.0)	7 (12.5)	49 (87.5)		0 (0.0)	7 (12.1)	51 (87.9)	
Non-relapse				31 (79.5)	8 (20.5)	0 (0.0)		32 (84.2)	6 (15.8)	0 (0.0)	
rs17220087	32813299	HLA-DOB, intron	rs2071479	A/A	A/C	C/C	0.7691	A/A	A/C	C/C	0.0599
Relapse				0 (0.0)	3 (21.4)	11 (78.6)		0 (0.0)	3 (20.0)	12 (80.0)	
Non-relapse				1 (2.6)	6 (15.4)	32 (82.1)		0 (0.0)	1 (2.6)	37 (97.4)	
rs2071479	32813335	HLA-DOB, intron	rs2071479	C/C	C/T	T/T	0.2663	C/C	C/T	T/T	0.2762
Relapse				13 (92.9)	1 (7.1)	0 (0.0)		14 (93.3)	1 (6.7)	0 (0.0)	
Non-relapse				39 (100.0)	0 (0.0)	0 (0.0)		38 (100.0)	0 (0.0)	0 (0.0)	
rs17213693	32813344	HLA-DOB, intron	rs2071479	C/C	C/G	G/G	0.6869	C/C	C/G	G/G	0.0637
Relapse				0 (0.0)	3 (21.4)	11 (78.6)		0 (0.0)	3 (20.0)	12 (80.0)	

Non-relapse				0 (0.0)	6 (15.4)	33 (84.6)		0 (0.0)	1 (2.6)	37 (97.4)	
rs2070121	32813777	HLA-DOB, exon	rs2071479	C/C	C/G	G/G	N/A[#]	C/C	C/G	G/G	N/A
Relapse				0 (0.0)	0 (0.0)	11 (100.0)		0 (0.0)	0 (0.0)	12 (100.0)	
Non-relapse				0 (0.0)	0 (0.0)	29 (100.0)		0 (0.0)	0 (0.0)	32 (100.0)	

[¥]Assembly version: GRCh37.p13. [§]The sequenced SNPs were selected and studied based on the transplant determinants identified by Petersdorf et al. (2013).
[$]UTR: untranslated region. [#]N/A: not available.
